# Accurate Wheat Lodging Extraction from Multi-Channel UAV Images Using a Lightweight Network Model

**DOI:** 10.3390/s21206826

**Published:** 2021-10-14

**Authors:** Baohua Yang, Yue Zhu, Shuaijun Zhou

**Affiliations:** 1 School of Information and Computer, Anhui Agricultural University, Hefei 230036, China; zhuyue@stu.ahau.edu.cn (Y.Z.); zhoushuaijun@stu.ahau.edu.cn (S.Z.); 2 Anhui Provincial Engineering Laboratory for Beidou Precision Agriculture Information, Anhui Agricultural University, Hefei 230036, China; 3 Smart Agriculture Research Institute, Anhui Agricultural University, Hefei 230036, China

**Keywords:** UAV, wheat lodging, deep learning, lightweight, digital surface model (DSM)

## Abstract

The extraction of wheat lodging is of great significance to post-disaster agricultural production management, disaster assessment and insurance subsidies. At present, the recognition of lodging wheat in the actual complex field environment still has low accuracy and poor real-time performance. To overcome this gap, first, four-channel fusion images, including RGB and DSM (digital surface model), as well as RGB and ExG (excess green), were constructed based on the RGB image acquired from unmanned aerial vehicle (UAV). Second, a Mobile U-Net model that combined a lightweight neural network with a depthwise separable convolution and U-Net model was proposed. Finally, three data sets (RGB, RGB + DSM and RGB + ExG) were used to train, verify, test and evaluate the proposed model. The results of the experiment showed that the overall accuracy of lodging recognition based on RGB + DSM reached 88.99%, which is 11.8% higher than that of original RGB and 6.2% higher than that of RGB + ExG. In addition, our proposed model was superior to typical deep learning frameworks in terms of model parameters, processing speed and segmentation accuracy. The optimized Mobile U-Net model reached 9.49 million parameters, which was 27.3% and 33.3% faster than the FCN and U-Net models, respectively. Furthermore, for RGB + DSM wheat lodging extraction, the overall accuracy of Mobile U-Net was improved by 24.3% and 15.3% compared with FCN and U-Net, respectively. Therefore, the Mobile U-Net model using RGB + DSM could extract wheat lodging with higher accuracy, fewer parameters and stronger robustness.

## 1. Introduction

Wheat is the main food source in the world, the quality and yield of which are related to food security [1]. Lodging is a common agricultural natural disaster in wheat production, especially in the middle and late stages of wheat growth, and it is one of the important factors that limit the high yield of wheat [2]. On the one hand, lodging changes the individual development of wheat and, on the other hand, lodging changes the population structure of wheat. Previous studies have shown that lodging not only affects protein synthesis and nutrient transport, but also causes a sharp decline in photosynthetic rate and dry matter production capacity [3]. Therefore, it is of great significance for production management, prevention and control guidance, as well as disaster assessment for agricultural departments and agricultural insurance departments, to accurately and quickly obtain information, such as the location and area of wheat lodging. 

The traditional method of obtaining lodging information is ground manual measurement, which is time-consuming and labor-intensive and its measurement results are subjectively affected. In addition, for large-scale lodging disasters, its low work efficiency often cannot meet actual needs [4]. In contrast, the rapid development based on remote sensing technology provides a practical means for large-scale and rapid monitoring of lodging information [5], such as near-ground remote sensing, satellite remote sensing and unmanned aerial vehicle (UAV) remote sensing monitoring. The low efficiency of near-ground remote sensing technology limits its further application on the farmland scale [6]. To achieve large-scale crop lodging monitoring, Yang et al. used the Radarsat-2 radar polarization index method to monitor wheat lodging [7]. Chauhan et al. used Sentinel 1 radar data and Sentinel 2 multispectral data to monitor the incidence of wheat lodging [8]. To make full use of the information provided by satellites, Chauhan et al. realized the classification of the degree of lodging of wheat by combining satellite data and the measured crop height on the ground [9]. However, for the limitation of time resolution, satellites cannot quickly obtain data to meet the needs of real-time identification. Therefore, it is necessary to develop a fast and reliable method for identifying wheat lodging. In recent years, UAV remote sensing has made up for the shortcomings of satellite remote sensing and near-ground remote sensing by virtue of its advantages of miniaturization, low cost, simple operation and high spatial and temporal resolution. UAV is the main tool for rapid and accurate acquisition of crop information in the application of agricultural quantitative remote sensing. UAV remote sensing is the current research hotspot and the future research trend. Previous studies have shown that remote sensing technology based on UAV can detect not only lodging in high-density crops, such as buckwheat [10], rice [11], barley [12], wheat [13] and jute [14], but low-density crop lodging information acquisition, such as corn [15], sunflower [16], cotton [17] and sugarcane [18], has also achieved good results. In addition, many scholars have also carried out analyses of crop lodging based on different features extracted by UAV, including spectral information [19], texture features [20], gray level co-occurrence matrix [21] and vegetation indices [22]. In any case, the above research papers showed the feasibility of extracting crop lodging based on digital images obtained from UAV. However, it is difficult to achieve accurate lodging detection tasks for traditional features. Therefore, it is expected that more robust features will be used to identify wheat lodging.

At present, UAV not only obtains digital images with three channels of R, G and B, but also can generate a variety of derivative models based on multiple aerial images, including digital orthophoto (DOM), digital elevation model (DEM) and digital surface model (DSM), which have been successfully used in the application of monitoring crop growth. Among them, DSM has received extensive attention because of its rich information and intuitive reflection of features such as canopy, location and height. Handique et al. used the difference of DSM to distinguish crops of different heights [23]. Feng et al. utilized DSM to successfully estimate crop yields [24]. In general, the DSM generated by UAV images can accurately represent the spatial variability of crops in different growth states. Yang et al. successfully realized the lodging detection of rice using DSM and texture features generated by UAV images [25]. In fact, fusion images based on RGB images contain multi-channel information, which can provide more heterogeneous features for lodging recognition. For example, some studies have focused on fusion image combining RGB and DSM to extract lodging, while other studies have developed a method of fusing RGB and the vegetation index to extract lodging [26]. At present, there is no universally accepted understanding of which information is better to fuse aerial images obtained by UAV. In addition, most of the research was still based on manually extracted features. Therefore, the extraction of crop lodging information still faces many challenges.

With the enhancement of computer processing power, the recognition of crop lodging based on deep learning has become a research hotspot in the field of agriculture. Many methods based on convolutional neural networks have been successfully applied to the research of lodging recognition. Yang et al. used EDANet to extract the lodging information of rice [27]. Zhao et al. utilized U-Net to extract the lodging area of rice [28]. Compared with traditional algorithms, the advantage of deep learning is that it can automatically extract effective features through a multi-layer neural network. In particular, the convolutional neural network model not only extracts the local detailed features of the image, but also extracts the high-level semantic features of the image. Research results showed the feasibility and superiority of extracting crop lodging information based on deep learning. However, the limitations of large amounts of calculation and high resource consumption still make the model complex, which makes it difficult to meet the needs of large-scale, real-time detection. In particular, it was not known whether the multi-channel image of fusion information could further improve the accuracy of lodging information extraction. Although Li et al. exploited deep learning methods to achieve lodging area segmentation based on multi-channel spectral information [29], so far, it is not clear how fusion-based multi-channel images could detect crop lodging based on lightweight neural network models.

Therefore, a method for extracting wheat lodging information based on a light-weight U-Net model with depthwise separable convolution is proposed in this study. Self-built data sets obtained from UAV were used to evaluate the performance of the model, including RGB of three channels, RGB + DSM of four channels and RGB + ExG of four channels. The purpose of this research study is to (1) train Mobile U-Net using self-built data sets and fine-tune model parameters to improve the robustness of the model, (2) verify the effectiveness of the multi-channel fusion image to improve the accuracy of wheat lodging extraction and (3) compare ours with other models to evaluate the performance of the proposed model.

## 2. Materials and Methods

### 2.1. Data Collection

The field experiment was conducted in the National Modern Agriculture Demonstration Zone (31°29′26″ N, 117°13′46″ E) located in Guohe Town, Lujiang County, Anhui Province, China. The area belongs to the subtropical monsoon climate, with four distinct seasons, obvious cold and heat and it is suitable for the cultivation of wheat. Thirty-six plots in the experimental area were selected as the study area, each plot covering the area of 144.3 square meters (78 × 1.85 m^2^). The large row spacing was 0.3 m and the small row spacing was 0.1 m. The variety of wheat was ‘Wanmai 55’. From 30 April to 26 May 2021, Lujiang County experienced severe convective weather such as severe storms and rains, with winds reaching up to 7–8 levels, and severe weather such as hail in some areas, leading to multiple lodging of wheat in the study area. The wheat in the experimental area was in the critical period of wheat growth. During this period, members of our team collected UAV images and ground information at different stages of wheat growth, including the flowering (7 May 2021), filling (17 May 2021) and maturity (27 May 2021) stages. 

During the data collection process, a total of 298 UAV aerial images was obtained at a height of 30 m above the ground during the three growth stages of wheat, including flowering (98 images), filling (100 images) and maturity (100 images). The size of a single image was 4000 × 3000 pixels. The Pix4DMapper software (Pix4D, Prilly, Switzerland) was used to stitch the original images to obtain orthophotos of wheat fields in three periods. Then, the acquired aerial images were manually annotated, cropped and subjected to data augmentation. 

**Figure 1:**
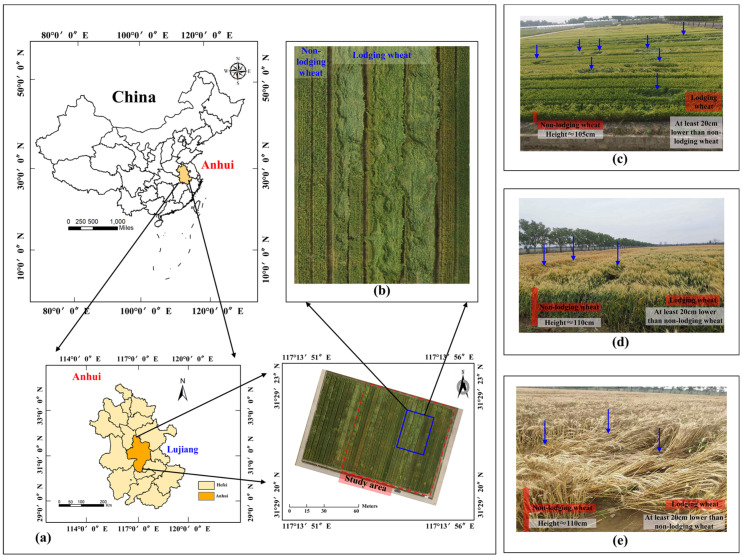
Location of UAV imaging area, study site and lodging samples: (**a**) study site; (**b**) partially enlarged display of the wheat field; the close-up maps of lodging and healthy wheat in (**c**) flowering, (**d**) filling and (**e**) maturity. The field indicated by the blue arrow is the lodging wheat.

Figure 1a shows the research location; Figure 1b is a partially enlarged display of the wheat field. It is easy to see that the lodging area was very large and the degree of lodging was very serious. Figure 1c shows a close-up map of lodging and healthy wheat in flowering stage; the image of the wheat field was acquired by UAV at a height of about 3 m above the ground and the shooting angle was about 65°. Figure 1d shows a close-up map of lodging and healthy wheat in filling stage, Figure 1e shows a close-up map of lodging and healthy wheat in maturity stage. We found that the height of lodging wheat is significantly lower than that of non-lodging wheat by at least 20 cm. Figure 1d,e was obtained using a mobile phone (nova5 pro, ISO: 50, focal length: 26 mm).

### 2.2. Data Preprocessing

#### 2.2.1. Image Annotation

**Figure 2:**
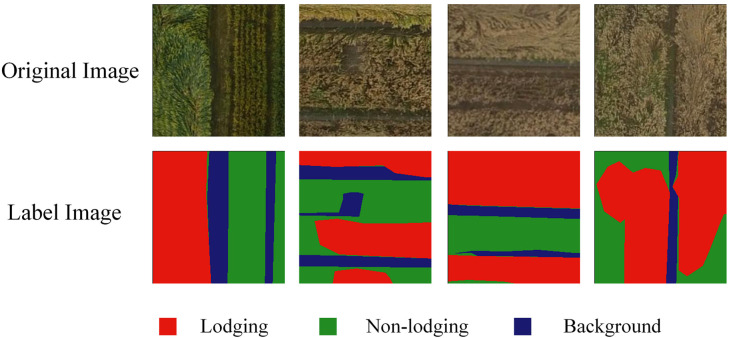
Example of original image and labeled image after cropping.

Among them, the Labelme software (http://labelme.csail.mit.edu/Release3.0/, accessed on 10 May 2021) was used to manually mark; the non-lodging area of wheat was marked as wheat, the lodging area was marked as lodging, the other areas were marked as background. The label images were created and the annotated images were cropped into images with 256 × 256 pixels, as shown in Figure 2.

#### 2.2.2. Image Fusion

To explore the influence of DSM and ExG on the recognition of the lodging effect based on the deep learning model, the RGB images collected by the UAV in this study were calculated to obtain the ExG index, the DSM was generated based on the dense point cloud and then the band was synthesized by the ENVI5.3 (Exelis Visual Information Solutions, USA) software. The ExG and DSM were added to the RGB image as the fourth band to obtain fusion images of RGB + ExG and RGB + DSM.

Among them, high-resolution, multi-view dense images were obtained from UAV and then Pix4Dmapper (Pix4D Company, Switzerland) software was used to adjust and match the images to generate dense point clouds; then, the triangulated irregular network (TIN) was constructed and, finally, a digital surface model (DSM) was obtained.

Excess green (ExG) can better distinguish vegetation and soil and it is often used for crop remote sensing monitoring [30]. To increase the extraction accuracy of wheat lodging information, the ENVI5.3 software was used to extract the gray values of the three bands of R, G and B from the RGB image obtained by UAV aerial photography and then the ExG index was calculated according to Equation (1).

(1) $$\mathrm{ExG}=\frac{2R-G-B}{R+G+B}$$

where G, B and R are the visible light green band, blue band and red band respectively.

#### 2.2.3. Image Augmentation

To obtain more training samples, data augmentation was performed on training sample images and label images. A lossless transformation method was used, i.e., random horizontal or vertical flipping, random rotation at 90° and random x–y coordinate axis transposition. Therefore, data sets based on RGB and fusion images (four channels based on RGB + ExG and four-channel images based on RGB + DSM) were constructed, each including 1500 images. Different lodging detection models were trained based on three different data sets, training sets, validation sets and test sets, which included 1200, 150 and 150 images, respectively.

### 2.3. Model Construction and Evaluation Indicators

#### 2.3.1. U-Net Model

U-Net is currently a popular deep learning model for semantic segmentation, which consists of a convolutional coding unit and a convolutional decoding unit [31]. Generally, the coding unit is mainly used to capture the context information in the image and the decoding unit is used to accurately locate the part that needs to be divided. Although the U-Net performance has been improved by improving the fully convolutional network (FCN), the standard U-Net neural network still needs to be further improved. To improve the detection accuracy, we proposed a wheat lodging recognition model combining MobileNetV1 with depthwise separable convolution and U-Net to form a wheat lodging segmentation model. 

#### 2.3.2. Mobile U-Net Model

**Figure 3:**
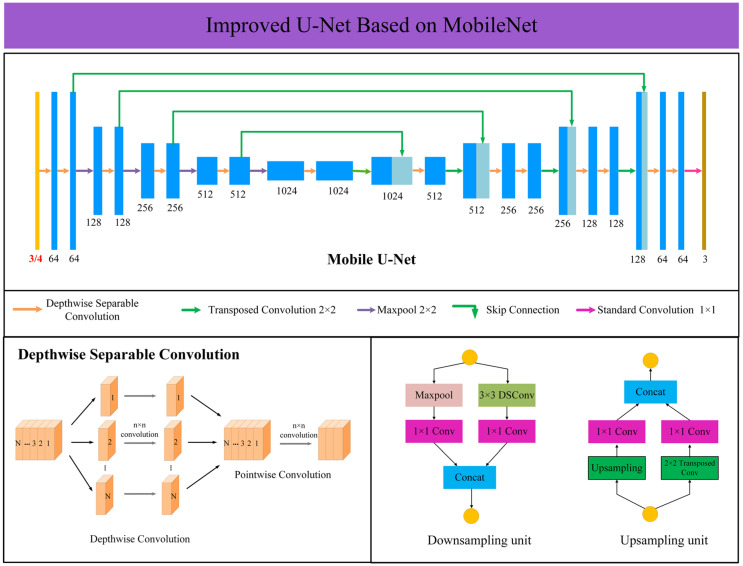
The structure of the Mobile U-Net model. (3/4 means that the parameter is set to 3 for RGB as input data and the parameter is set to 4 for four-channel image as input).

The Mobile U-Net model was composed of an encoder and a decoder. The ordinary convolution was replaced with a depthwise separable convolution to reduce the number of parameters and calculations of the entire network [32]. Among them, the pooling layer (Max pooling) and the convolutional layer were combined to construct a down-sampling unit, while the up-sampling layer and the convolutional layer were combined to construct an up-sampling unit. At the same time, depthwise separable convolution was used for feature extraction in the down-sampling unit, which enhances the feature extraction capability of the network model and reduces the computational cost. The addition of the convolutional layer could make up for the shortcomings of the Max Pooling layer and up-sampling layer that are not trainable, so it could reduce the loss of feature information during the sampling process and effectively improve the segmentation accuracy of the small boundary of the lodging edge of wheat, as shown in Figure 3.

The input of the model was an image with a resolution of 256 × 256 pixels (3-channel image or 4-channel image) and the output was a single-channel segmented image. In the convolutional coding unit, a total of 4 up-samplings was performed and the first up-sampling unit included 2 repeated depthwise separable convolution modules and a Max pooling layer. The second, third and fourth up-sampling units had the same structure, including a depthwise separable convolution module and a Max pooling layer. After each pooling operation, the feature map size decreased and the number of channels doubled. The decoder performed down-sampling through transposed convolution and gradually restored image information. Corresponding to the encoder part, the decoder performed a total of 4 down-samplings. The first down-sampling unit included a depthwise separable convolution module and a transposed convolution module. The second, third and fourth down-sampling units also had the same structure, including two repeated depthwise separable convolution modules and one transposed convolution module, respectively. Each up-sampling expanded the feature map size and reduced the number of channels by half. Finally, a standard convolution module with a size of 1 × 1 was used to reduce the dimension and a normalized exponential function (SoftMax) was used to convert the value into a probability. The specific parameters are shown in the Table 1.

#### 2.3.3. Wheat Lodging Segmentation Model

**Figure 4:**
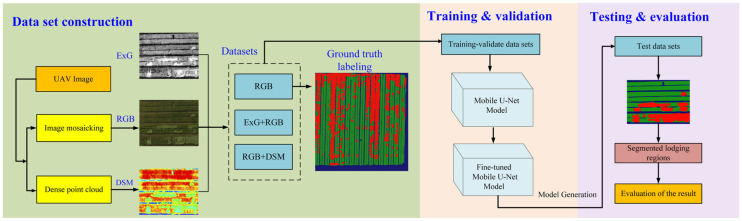
The technical flow chart of this study.

The technical process of this research study, shown in Figure 4, mainly included UAV digital image collection, data set construction, model training and verification, testing, model evaluation and optimization. Firstly, the DSM and ExG derived from the RGB image obtained by UAV were used to construct the RGB, RGB + ExG and RGB + DSM data sets. Secondly, the Mobile U-Net model proposed in this study was trained, verified and tested using different data sets. Furthermore, we compare the performance of Mobile U-Net with typical deep learning frameworks, such as FCN and U-Net. Finally, three data sets in different periods were used to predict the lodging area.

#### 2.3.4. Evaluation Indicators

There were four indicators used to evaluate the performance of the model, including precision, recall, F1-score and mean Intersection over Union (mIoU). Among them, precision shows the proportion of samples that are predicted to be lodging wheat in the segmented image that are actually lodging wheat; recall shows to the proportion of samples that are predicted to be lodging wheat among all the samples that are actually lodging wheat; F1-score is the harmonic mean of accuracy and recall, reflecting the comprehensive performance of segmentation of lodging wheat in the wheat field; mIoU is the ratio of overlap between the segmentation result of wheat lodging and ground truth. The values of the above evaluation indicators are all between 0 and 1 and the larger the value, the better the segmentation effect. In this study, precision, recall, F1-score and mIoU are used as the evaluation indexes for evaluating the segmentation accuracy of lodging wheat and the calculation formulas are as follows:

(2) $$precision=\frac{TP}{TP+FP}$$

(3) $$recall=\frac{TP}{TP+FN}$$

(4) $$F1-score=2\times \frac{precision\times recall}{precision+recall}$$

(5) $$mIoU=\frac{1}{k+1}\sum_{i=0}^{k}\frac{TP}{FN+FP+TP}$$

where TP refers to the correct segmentation of the wheat lodging area, which is the wheat lodging area; TN refers to the correct segmentation of the non-lodging area of wheat, which is a non-lodging area of wheat; FP refers to the correct segmentation of the wheat lodging area, which is a non-lodging area of wheat; FN refers to the correct segmentation of the non-lodging area of wheat, which is the wheat lodging area; k is the number of categories.

## 3. Results

### 3.1. DSM and ExG Images Derived from RGB 

**Figure 5:**
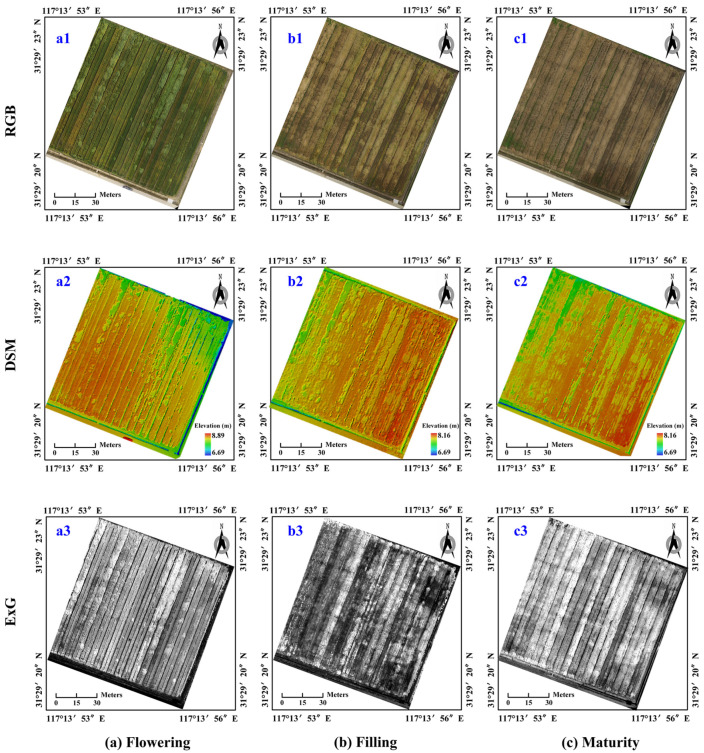
RGB, DSM and ExG of wheat fields in different growth periods: (**a**) flowering, (**b**) filling and (**c**) maturity.

Pix4Dmapper was used to generate a high-precision DSM (digital surface model) and ExG (excess green) in the wheat research area with high-resolution digital images obtained from UAV in different growth periods, as shown in Figure 5. Among them, the first column represents the flowering period, the second column represents the filling period and the third column represents the maturity period, as shown in Figure 1a–c. The first row represents the RGB image of the study area, the second row represents the DSM extracted from the image of the study area and the third row represents the ExG extracted from the image of the study area.

It can be seen, from Figure 5 (a2, DSM of flowering period; b2, DSM of filling period; c2, DSM of maturity period), that the elevations of the digital surface models in different periods were still significantly different. Especially, in the same period, the elevation of the wheat field was also different, because the digital surface model covered the elevation of other surface information except the ground. In this study, DSM showed the ground elevation model of normal wheat and lodging wheat, which could most truly express the growth status of crops on the ground of wheat fields. Therefore, DSM was beneficial to distinguish between normal wheat and lodging wheat in the field.

In addition, to clarify the contribution of the ExG index in identifying lodging wheat, the digital numbers (DNs) of the R, G and B channels were extracted from the RGB images of the study area acquired in three different periods; then, ExG was calculated and the visualization of ExG is shown in Figure 5 (a3, ExG of flowering period; b3, ExG of filling period; c3, ExG of maturity period). It can be seen, from Figure 5, that ExG was different in different periods. 

**Figure 6:**
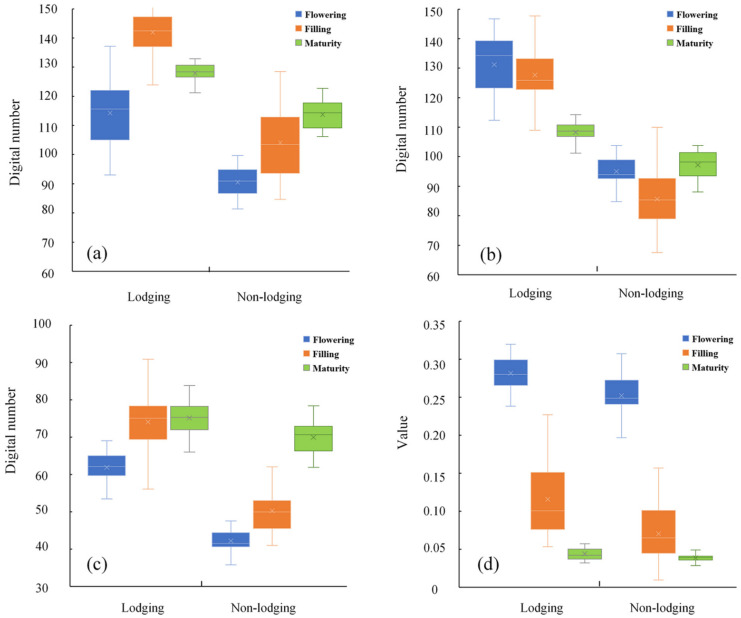
Comparison of the ExG values of lodging and non-lodging in different periods: (**a**) digital number of R; (**b**) digital number of G; (**c**) digital number of B; (**d**) value of ExG.

Figure 6a–d shows the specific values of the digital number of R, digital number of G, digital number of B and ExG of lodging and non-lodging wheat in different periods extracted from the set 30 regions of interest (ROI). It can be seen, from Figure 6a–d, that the distribution of R, G, B and ExG was different in the flowering, filling and maturity periods. Especially, Figure 6d shows that the ExG of non-lodging wheat was significantly lower than that of lodging wheat. The mean values of ExG were 0.193–0.307, 0.009–0.157 and 0.027–0.049 for non-lodging and 0.238–0.319, 0.053–0.227 and 0.032–0.07 for lodging at the flowering, filling and maturity stage, respectively. Among them, the average ExG values of lodging wheat fields were 0.281, 0.116 and 0.044 in the three periods, which were 10%, 39% and 12% higher than those of normal wheat fields. It can be seen that ExG had a positive effect on the identification of wheat lodging.

### 3.2. Model Parameter Setting and Training

The experimental environment of this research project was the Windows10 Professional 64-bit operating system and the deep learning framework was Keras 2.2.4, which was used to train the network model. Model training and verification environment were as follows: Intel(R) Core (TM) i7-8700 @3.20 GHz and 16 G NVIDIA GeForce RTX 2080. The images were stitched with Pix4Dmapper and were cropped with Python codes. The language of model development used was python.

**Figure 7:**
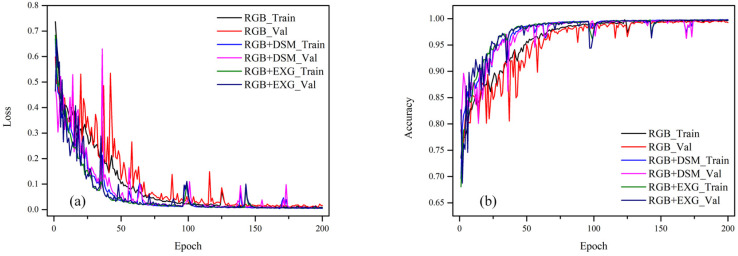
Loss and accuracy curves of the training set and validation set: (**a**) loss curve; (**b**) accuracy curve of training set and verification set. Train indicates training set; Val indicates validation set.

The model was trained using the Adam algorithm, the learning rate was 0.0001, the Batch size was 4 and the training iterations were 200 Epochs. After each Epoch training, not only the loss and accuracy were obtained by calculation, but the weights were also updated and saved. After the model was trained for 200 Epochs, the model with the highest accuracy was selected as the test model. Figure 7 shows the loss and accuracy curves of the training set and the validation set (RGB, RGB + ExG and RGB + DSM) of the Mobile U-Net model. It can be seen, from Figure 7, that that the error between the training set and the validation set decreased with the increase in the number of iterations and the error dropped below 0.1 when epoch = 65, then finally stabilized. On the one hand, this shows that the model can control the deviation. However, the close error of the training set and the verification set after stabilization indicated that the variance of the model was relatively low. In addition, the accuracy of the network increased as the number of iterations increased, until it stabilized. Therefore, when the training converged, the model with the highest accuracy was selected as the test model.

### 3.3. Results of Wheat Lodging Recognition with Different Data Sets

Table 2 showed the test results of data sets for different growth periods based on the Mobile U-Net model. Among them, the F1-score of the training set was 74.31–94.87% and the mIoU was 70.21–91.31%. The F1-score of the test set was 70.45–96.82% and the mIoU was 62.11–87.99%. Therefore, the Mobile U-Net model performed well in the extraction of wheat lodging. In particular, the F1-score of wheat lodging segmentation was 70.45–85.42% for RGB, 78.49–90.37% for RGB + ExG and 80.8–96.82% for RGB + DSM. The corresponding mIoU were 62.11–74.68%, 69.58–83.45% and 70.39–87.99%.

**Figure 8:**
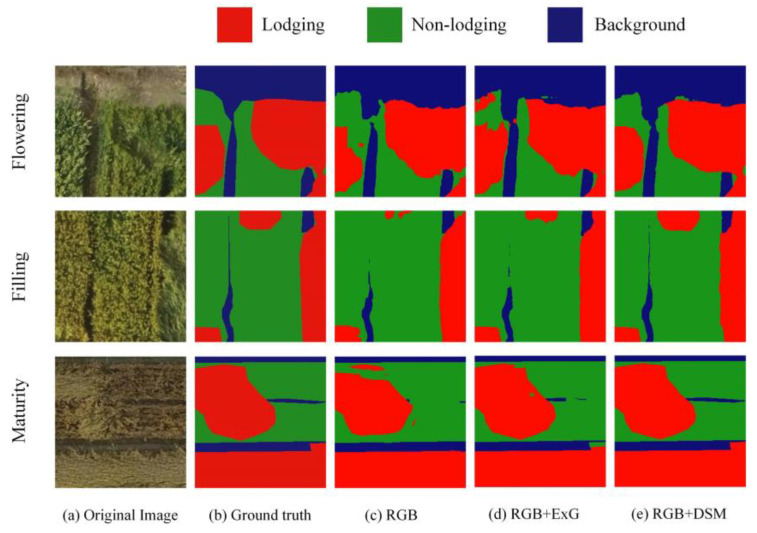
Test results of lodging recognition using different data.

Figure 8 shows the lodging segmentation results of three different data sets in different periods, including RGB, RGB + ExG and RGB + DSM. It could be seen from Figure 8a that the lodging degree of wheat in the three different periods was quite different and the canopy structure was also different. Figure 8b represents the ground truth of wheat lodging. Figure 8c–e shows the results of wheat lodging recognition. Among them, the lodging recognition error rate with the RGB image was relatively high. There were many missed recognitions in lodging recognition using RGB + ExG. The result of lodging recognition using RGB + DSM was close to ground truth.

## 4. Discussions

### 4.1. Compare the Identifying Results of Wheat Lodging Using Different Fusion Images

The visible light vegetation index could quantify the growth of vegetation under certain conditions, because it could reflect the difference between the reflection of vegetation under visible light and the soil background [33]. Some studies have used the vegetation index to successfully extract crop lodging information. For example, Wu et al. used NDVI to extract the lodging of rice [34]. Zhao et al. used a combination of three vegetation indices, including super green (ExG), super red (ExR) and the visible band difference vegetation index (VDVI), to successfully extract the lodging area of rice [28]. They only carried out the lodging extraction study based on the spectral characteristics of the vegetation, but did not carry out the comparison with the image fusion. In particular, the identification of lodging and non-lodging based only on the spectral characteristics of the wheat canopy could easily lead to misidentification and low recognition accuracy, because it was inevitable that the same objects had different spectra and the same spectrum reflected different objects. Therefore, it was necessary to study the different characteristics of the canopy in order to improve the accuracy of lodging detection.

In this study, three wheat field data sets of different growth periods were constructed, including RGB, RGB + ExG and RGB + DSM. Table 3 shows the comparison of the lodging recognition results based on data sets in different periods. Compared with RGB, F1-score and mIoU based on RGB + DSM increased by 12.8% and 11.8% in the flowering stage, increased by 11.8% and 15.1% in the filling stage and increased by 10.9% and 12.5% in the maturity period. In the corresponding period, F1-score and mIoU were 2.9% and 1.2%, 6.7% and 5.2%, and 8.7% and 9.6% higher than that of RGB + ExG, respectively. It is worth mentioning that there were significant differences in the elevations displayed in the DSM of the study area before and after the lodging of the wheat. Therefore, DSM fully expressed the difference in elevation between ground features in different periods, which was suitable for distinguishing lodging wheat from normal wheat.

### 4.2. Compare the Identifying Results of Wheat Lodging Based on Different Methods

To further verify the performance of our proposed method, the classic segmentation method U-Net model and FCN model under the deep learning framework were selected and compared with the model proposed in this paper on three identical test sets (150 images). The hardware environment for model testing was Intel(R) Core (TM) i7-1065G7 @1.30 GHz, 16 G. The results are shown in Table 3. It can be seen, from Table 3, that, compared with FCN and U-Net, the F1-Score of Mobile U-Net increased by 24.3% and 15.7% and mIoU increased by 17.7% and 12.5% for the RGB; 25.9% and 17.3% of F1-Score, 28.7% and 20.6% of mIoU for the RGB + ExG; 24.3% and 15.3% of F1-Score, 30.7% and 19.5% of mIoU for the RGB + DSM. Therefore, regardless of RGB, or the fused image RGB + ExG and RGB + DSM, the Mobile U-Net proposed in this study was superior to FCN and U-Net in wheat lodging recognition. In particular, F1-Score and mIoU based on Mobile U-Net using RGB + DSM was 88.99%, 80.7%, 11.8% and 14.3% higher than that of RGB and 6.2% and 6.7% higher than that of RGB + ExG.

Table 3 shows the comparison results of the average time for different models to process each image. After the model was tested using the test sets, the average time for Mobile U-Net to process each four-channel image with a size of 256 × 256 was 0.53 s using CPU (Intel(R) Core (TM) i7-1065G7, @1.30 GHz, 16 G). Both U-Net and FCN took longer to process the same types of images than the Mobile U-Net model. Regarding processing time per image, Mobile U-Net was 37.5% and 44.4% faster than U-Net and FCN for RGB, 23.8% and 33.3% faster for RGB + ExG, and 27.3% and 33.3% faster for RGB + DSM. In addition, regarding the parameters of the model, FCN was 17.08 million, U-Net was 30.95 million and Mobile U-Net was only 9.49 million, which was the model with the fewest parameters among the three models. Therefore, the model proposed in this study ensured that the accuracy was not reduced and improved the speed of image segmentation, aiming to achieve the goal of early warning of wheat lodging, reducing the impact of lodging, increasing production and income and benefiting farmers. 

In fact, some studies have shown that semantic segmentation methods based on deep learning have strong advantages in lodging recognition. Yang [26] et al. used FCN (full neural network) to extract rice lodging based on RGB + ExG fusion information. Zhao et al. used UNet to extract lodging information [28]. Although the above-mentioned deep learning methods could effectively extract lodging features, too many parameters resulted in a low operating speed of the model. The possible reason is that the structure of the model they adopted was more complicated. For example, the standard U-Net neural network consists of 19 convolutional layers, the corresponding pooling layers and up-sampling layers. Therefore, it was necessary to improve the model, aiming to reduce the amount of calculation and improve the recognition effect.

**Figure 9:**
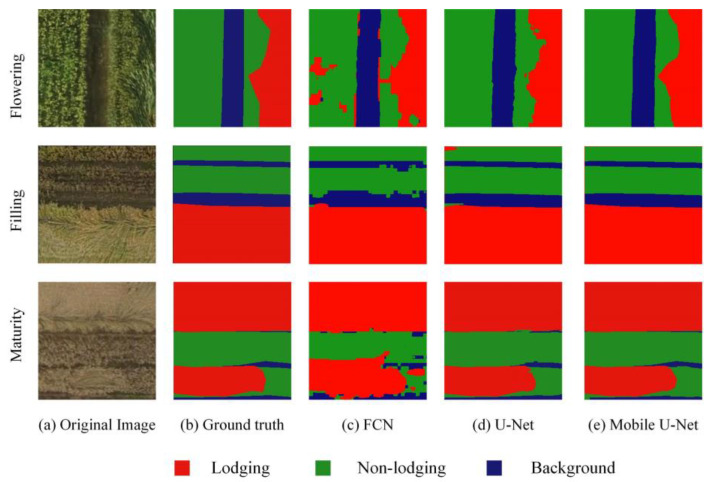
The results of estimating wheat lodging with different models with RGB + DSM.

To show the recognition of wheat lodging based on different models, only the recognition results using the RGB + DSM were provided here, as shown in Figure 9. It can be seen, from Figure 9, that there were many wrong recognitions based on the FCN. The lodging detection based on U-net was close to the result of our method and there were still some areas missing recognition. According to the analysis in Table 3, compared with FCN and U-net, the model we proposed not only maintained the premise of the same accuracy, but also improved the processing speed and reduced the parameters of the model, providing a technical basis for portable mobile devices that detect lodging in the field.

### 4.3. Visualization of Feature Activation in Lodging Wheat

To verify the function of the depthwise separable convolution module, gradient-weighted class activation mapping (Grad-CAM) [35], which mainly uses the gradient of the target class and propagates to the final convolutional layer to generate a rough positioning map, was used to visualize the features,. The results of visualization clearly show how the network model selects important areas of the prediction class, so as to determine the impact of the depthwise separable convolution module.

**Figure 10:**
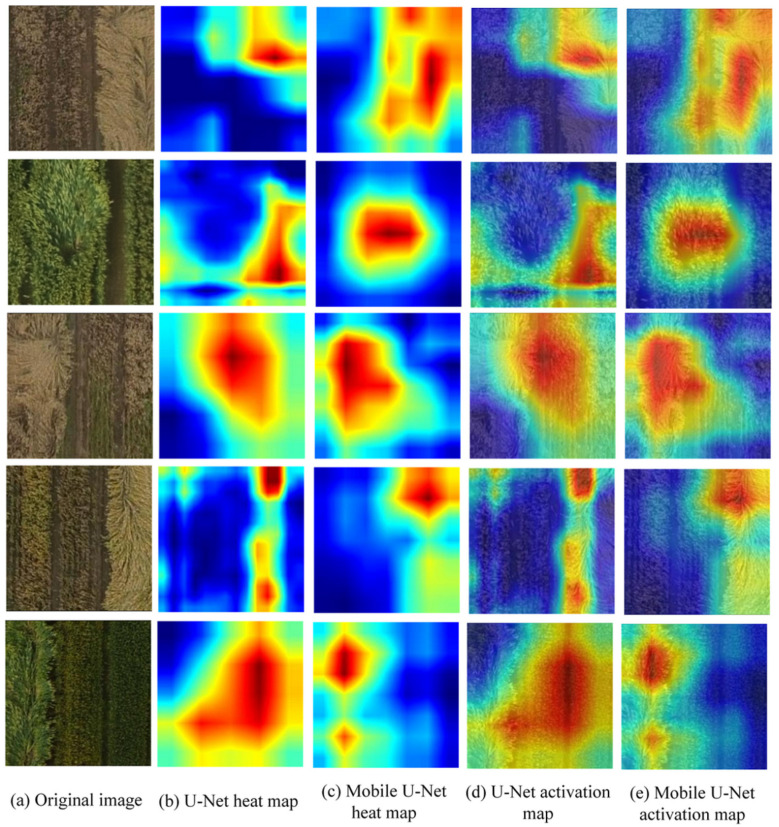
Visualization of feature activations using Grad-CAM.

As shown in Figure 10, the red area in the feature map indicates the high-weight area of the neural network to determine the lodging wheat and the blue area indicates the low-weight area of the network to determine the lodging wheat. The redder the color, the greater the influence of this area on the recognition result of the lodging wheat. It can be seen, from Figure 10b,d, that U-Net focused on the lodging area, non-lodging area and background. Figure 10c,e shows that the Mobile U-Net model paid attention to the more accurate lodging areas. Therefore, our proposed model with a depthwise separable convolution module could better learn the characteristic information of lodging wheat and improve the segmentation accuracy of lodging wheat.

## 5. Conclusions

In this study, a wheat lodging segmentation model based on a lightweight U-Net neural network with depthwise separable convolution, which was used to realize wheat lodging recognition and accurate segmentation from UAV images under field conditions, is proposed. The proposed model was trained, verified and tested with self-built wheat data sets (RGB, RGB + ExG, RGB + DSM) of different growth periods, including flowering, filling and maturity. The experiments showed that the extraction of wheat lodging effect based on the fusion image of DSM and RGB was the best; the F1-Score reached 88.99% and the mIoU reached 80.7%, indicating that the fusion image was more suitable for wheat lodging extraction. Furthermore, the parameters of the Mobile U-Net model were 9.49 million and the overall accuracy of Mobile U-Net was improved by 24.3% and 15.3% compared with FCN and U-Net, which indicate that the proposed model was suitable for the task of quickly and accurately detecting wheat lodging in the field.

## Figures and Tables

**Table 1:** Parameters of the Mobile U-Net model.

Layer Type	Size	Filter	Stride
Input	256 × 256 × 3/4		
Depthwise separable convolution	256 × 256 × 64	3 × 3, 1 × 1	2
Max pooling	128 × 128 × 64	2 × 2	1
Depthwise separable convolution	128 × 128 × 128	3 × 3, 1 × 1	2
Max pooling	64 × 64 × 128	2 × 2	1
Depthwise separable convolution	64 × 64 × 256	3 × 3, 1 × 1	2
Max pooling	32 × 32 × 256	2 × 2	1
Depthwise separable convolution	32 × 32 × 512	3 × 3, 1 × 1	2
Max pooling	16 × 16 × 512	2 × 2	1
Depthwise separable convolution	16 × 16 × 1024	3 × 3, 1 × 1	2
Transposed Convolution	32 × 32 × 512	3 × 3	1
Skip connection	32 × 32 × 1024		1
Depthwise separable convolution	32 × 32 × 512	3 × 3, 1 × 1	2
Transposed Convolution	64 × 64 × 256	3 × 3	1
Skip connection	64 × 64 × 512		1
Depthwise separable convolution	64 × 64 × 256	3 × 3, 1 × 1	2
Transposed Convolution	128 × 128 × 128	3 × 3	1
Skip connection	128 × 128 × 256		1
Depthwise separable convolution	128 × 128 × 128	3 × 3, 1 × 1	2
Transposed Convolution	256 × 256 × 64	3 × 3	1
Skip connection	256 × 256 × 128		1
Depthwise separable convolution	256 × 256 × 64	3 × 3, 1 × 1	2
Standard convolution	256 × 256 × 3	1 × 1	1

**Table 2:** Segmentation results using different data of three different periods.

Dataset		*F1-Score* (%)	*mIoU* (%)	*F1-Score* (%)	*mIoU* (%)
		Training Set		Test Set	
RGB	Flowering	74.31	70.21	70.45	62.11
	Filling	88.02	77.67	85.42	74.68
	Maturity	83.46	72.89	79.65	70.64
RGB + ExG	Flowering	81.32	76.53	78.49	69.58
	Filling	94.87	87.04	90.37	83.45
	Maturity	88.36	83.87	81.58	72.94
RGB + DSM	Flowering	89.69	85.94	80.8	70.39
	Filling	97.59	91.31	96.82	87.99
	Maturity	90.62	84.55	89.36	80.73

**Table 3:** Comparison of lodging recognition results of different models.

Methods	Data	*F1-Score*	*mIoU*	Time-CPU (s/Image)	Parameter (Million)
FCN	RGB	59.45	56.87	0.53	17.08
	RGB + ExG	61.90	53.72	0.70	17.08
	RGB + DSM	67.33	55.89	0.73	17.08
U-Net	RGB	66.17	60.51	0.60	30.95
	RGB + ExG	69.06	59.78	0.80	30.95
	RGB + DSM	75.36	64.95	0.80	30.95
Mobile U-Net	RGB	78.51	69.14	0.33	9.49
	RGB + ExG	83.48	75.32	0.53	9.49
	RGB + DSM	88.99	80.7	0.53	9.49

## Data Availability

Restrictions apply to the availability of these data. Data were obtained from the Smart Agriculture Research Institute of Anhui Agricultural University and are available from the authors with the permission of the Smart Agriculture Research Institute.
